# Investigation of an Influenza A (H3N2) outbreak in evacuation centres following the Great East Japan earthquake, 2011

**DOI:** 10.1186/1471-2458-14-34

**Published:** 2014-01-14

**Authors:** Taro Kamigaki, Jin Seino, Kentaro Tohma, Nao Nukiwa-Soma, Kanako Otani, Hitoshi Oshitani

**Affiliations:** 1 Department of Virology, Tohoku University Graduate School of Medicine, 2-1 Seiryo machi, Aoba-ku, Sendai 9808575, Japan; 2 National Hospital Organization Miyagi National Hospital, Yamamoto, Miyagi, Japan

**Keywords:** Influenza, Outbreak, Evacuation centre, Earthquake, Epidemiology

## Abstract

**Background:**

The Great East Japan Earthquake of magnitude 9.0 that struck on 11 March 2011 resulted in more than 18000 deaths or cases of missing persons. The large-scale tsunami that followed the earthquake devastated many coastal areas of the Tohoku region, including Miyagi Prefecture, and many residents of the tsunami-affected areas were compelled to reside in evacuation centres (ECs). In Japan, seasonal influenza epidemics usually occur between December and March. At the time of the Great East Japan Earthquake on 11 March 2011, influenza A (H3N2) was still circulating and there was a heightened concern regarding severe outbreaks due to influenza A (H3N2).

**Methods:**

After local hospital staff and public health nurses detected influenza cases among the evacuees, an outbreak investigation was conducted in five ECs that had reported at least one influenza case from 23 March to 11 April 2011. Cases were confirmed by point-of-care tests and those residues were obtained and subjected to reverse transcription PCR and/or real time RT-PCR for sub-typing of influenza.

**Results:**

There were 105 confirmed cases detected during the study period with a mean attack rate of 5.3% (range, 0.8%–11.1%). An epidemiological tree for two ECs demonstrated same-room and familial links that accounted for 88.5% of cases. The majority of cases occurred in those aged 15-64 years, who were likely to have engaged in search and rescue activities. No deaths were reported in this outbreak. Familial link accounted for on average 40.5% of influenza cases in two ECs and rooms where two or more cases were reported accounted for on average 85% in those ECs. A combination of preventative measures, including case cohorting, personal hygiene, wearing masks, and early detection and treatment, were implemented during the outbreak period.

**Conclusions:**

Influenza can cause outbreaks in a disaster setting when the disaster occurs during an epidemic influenza season. The transmission route is more likely to be associated with sharing room and space and with familial links. The importance of influenza surveillance and early treatments should be emphasized in EC settings for implementing preventive control measures.

## Background

The Great East Japan Earthquake of magnitude 9.0 that struck on 11 March 2011 resulted in more than 18000 deaths or cases of missing persons [1]. The large-scale tsunami that followed the earthquake devastated many coastal areas of the Tohoku region, including Miyagi Prefecture, and many residents of the tsunami-affected areas were compelled to reside in evacuation centres (ECs). Reportedly, more than 1300 ECs were established to accommodate more than 315000 evacuees in Miyagi Prefecture [2]. These ECs included officially designated centres and other buildings such as community centres, city halls, nursing homes, and schools to manage the huge surge of evacuees.

Japan has one of the most rapidly aging populations and the 2010 National Census found that 23% of the Japanese population was 65 years or older [3]. The coastal area of the Tohoku region, which was most severely affected by the tsunami, has a higher percentage of elderly residents than the national average and, consequently, an increase in the incidence of some diseases that affect the elderly, such as pneumonia [4], occurred after the earthquake.

After a natural disaster, the risk of infectious disease outbreaks often becomes a major concern. Infectious diseases that may cause outbreaks in post-disaster settings can be categorized into four groups: waterborne diseases, acute respiratory infections, vector-borne diseases, and infections as a result of wounds or injuries [5]. Of these diseases, acute respiratory infections, including influenza, are among the most common that occur after natural disasters [6,7]. However, there is limited information regarding influenza outbreaks after natural disasters [8] partly because of the limited ability to confirm suspected cases [7]. The influenza virus is one of the most common causes of acute respiratory diseases that are usually self-limited but can sometimes lead to severe complications such as pneumonia and influenza associated encephalopathy [9]. In Japan, influenza has been monitored under national surveillance with approximately 5,000 sentinel sites [10] and the estimated number of influenza outpatients was higher among children less than 10 years while approximately 45% of reported influenza inpatients were the elderly aged over 70 years in 2011/2012 season [11]. Residents aged ≥65 years are prioritized for influenza vaccination; 51% of the elderly of that age was vaccinated in 2006, which has been increased from 17% in 2000 [12]. In Japan, seasonal influenza epidemics usually occur between December and March. At the time of the Great East Japan Earthquake on 11 March 2011, influenza A (H3N2) was still circulating, and there was an increasing trend of influenza B toward April 2011, both nationwide [13] and in the affected areas [14,15]. Studies regarding influenza-associated mortality revealed that a higher impact was associated with influenza A (H3N2) compared with other seasonal influenza viruses [16,17] and influenza A(H1N1)pdm09 during the pandemic period [18], particularly among the elderly. Because the majority of evacuees in the ECs were elderly, there was a heightened concern regarding severe outbreaks due to influenza A (H3N2). To respond to a potential influenza outbreak in the ECs, we conducted an outbreak investigation in ECs located in Yamamoto, Miyagi Prefecture, Japan. In this study, we describe the epidemiological characteristics of influenza in an EC setting.

## Methods

### Study areas

Yamamoto is located on the southern coast of Miyagi Prefecture, and according to the 2010 National Census, it has a population of 16704, of which 31.6% are ≥65 years and 10.1% are ≤15 years [3]. Because of the devastating damage caused by the tsunami, a total of 634 people died or were missing and more than 5800 evacuees sought refuge in 19 ECs, 4 days after the earthquake.

### Outbreak investigation

After local hospital staff and public health nurses detected influenza cases among the evacuees, an outbreak investigation was conducted in five ECs that had reported at least one influenza case from 23 March to 11 April 2011. Two ECs were schools [EC (C) and (E)] and three were community centres [EC (A), (B) and (D)]. A febrile patient was initially identified by either self-reporting or health check done by public health nurses. The patient was seamlessly examined with the point-of-care (POC) test for influenza confirmation at either temporary clinic set up at evacuation centres or consultation rounds done by medical aid team. Due to the limited capacity to store and transport samples during that period, partial of residues obtained from the POC tests were subjected to reverse transcription PCR (RT-PCR) for influenza sub-typing. Epidemiological data, including demographic characteristics, residence, date of onset, clinical symptoms, familial links to previous cases, and history of wearing masks, were obtained by interviewing patients. A familial link was defined as a link between family members living in the same home before the earthquake. Data regarding the age distribution of evacuees could be obtained from only two ECs.

### Statistical analysis

An attack rate (AR) was defined as the proportion of confirmed influenza cases in the average number of evacuees in each EC on 25 March, 3 April and 10 April 2011. One EC could not count the number of evacuees on either 3 April or 10 April 2011 because it was merged with another EC. All data were compiled with Microsoft Excel (Microsoft Corp., Redmond, WA, USA) and analyzed with R 2.14.2 [19].

### Ethical consideration

An investigator explained the objectives of this investigation and obtained the verbal consent to participate this investigation from cases or parents or guardians if a case was children. The questionnaire was never completed if cases declined to participate. This study was reviewed and approved by the ethical committee of Tohoku University Graduate School of Medicine (2013-1-252).

## Results

**Figure 1:**
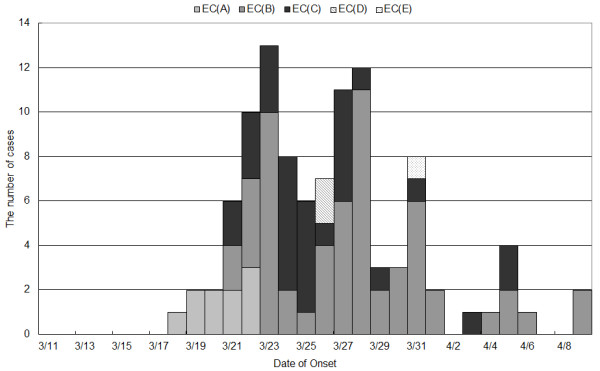
**The daily number of influenza cases in 5 evacuation centres, Yamamoto from 11 March to 9 April 2013.** Date of onset of a case from EC (B) and EC (E) was unknown. *EC, evacuation centre.

**Figure 2:**
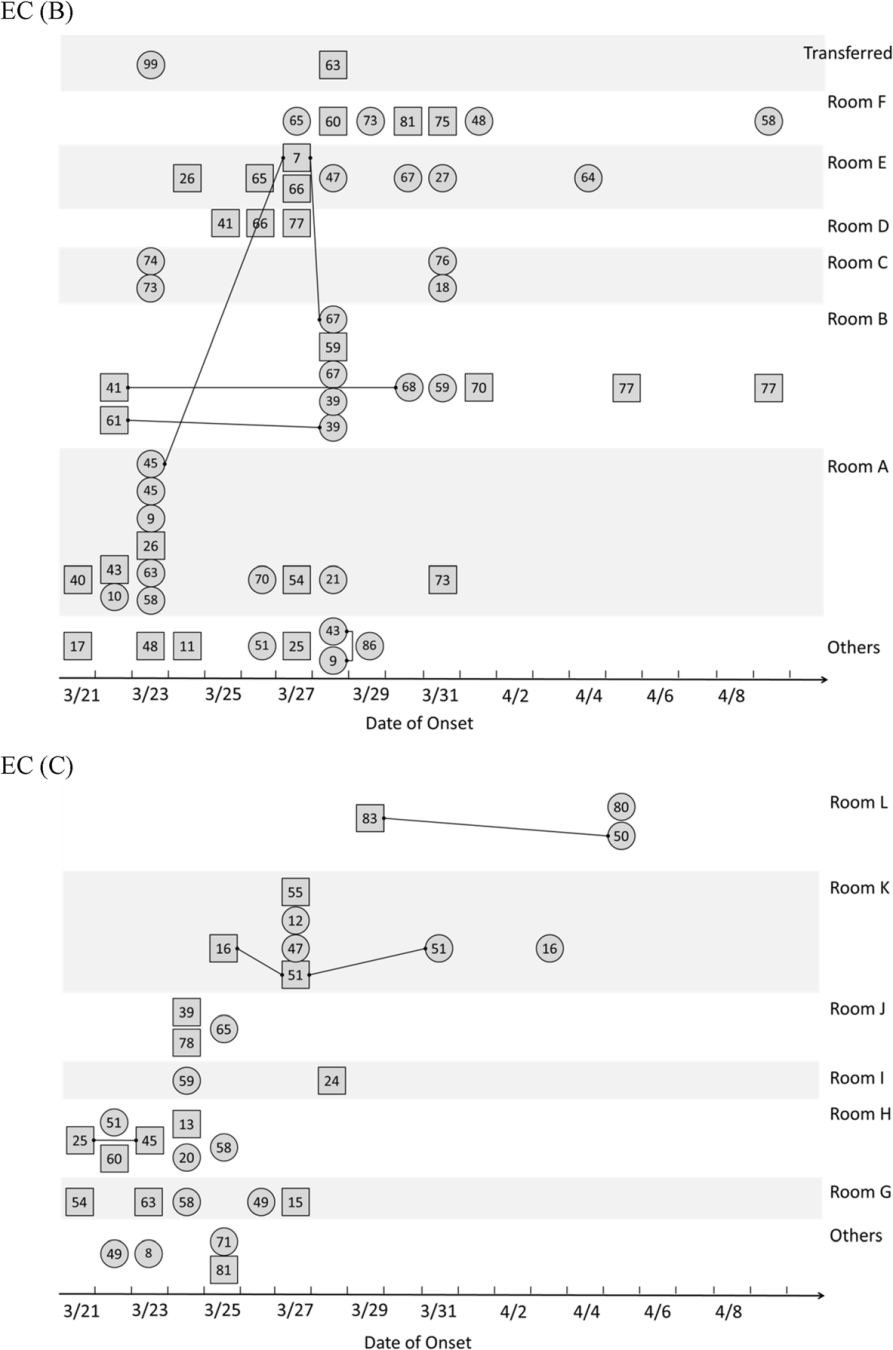
**Locations of influenza cases in the rooms and spaces of evacuation centre B (top) and C (bottom).** Encircled number indicated the age of cases. Rectangle and circle indicated male and female respectively. A line indicated a familiar link. Cases were categorized as others if the reported number in each room were less than 2. Cases who stayed outside were not included.

During this study period, 105 influenza cases were confirmed as influenza A using POC tests. In total, 27 residues of the POC tests were retested using RT-PCR, which further confirmed 22 positive influenza A (H3N2) cases. No influenza B cases were detected during the study period. The first confirmed case was identified in EC (A) on 18 March 2011 (post-disaster day 7), and further cases were reported from five additional ECs (Figure 1). There were two observed outbreak peaks on 23 March and 28 March 2011 (post-disaster days 12 and 17, respectively). During the study period, EC (B) recorded 60 influenza-positive cases which accounted for 57.1% of total cases [60/105], whereas EC (C) reported 31 cases, EC (A) reported 10 cases, and EC (D) and (E), where the first influenza cases were detected at later dates, reported only two cases each (Table 1). The highest overall AR (8.6%) was recorded in EC (B) followed by EC (A) (7.7%) and EC (C) (5.1%). The index cases for EC (A), (B) and (C) were adult males, whereas those in EC (D) and (E) were an adult female and a teenage male, respectively (Table 1). Overall, the male-to-female ratio of confirmed cases was 0.88. In EC (B) and (C), 36.3% and 28.7% residents, respectively, were aged ≥65 years. Thus, the AR for ≥65 years was determined as 12.2% in EC (B) and 3.4% in EC (C). ARs for <15 years and 15–64 years were 7.7% and 11.2% in EC (B), respectively, and 4.9% and 5.9% in EC (C), respectively. In EC (C), ARs for ≥65 years and ≤15 years were less than the overall AR. No influenza-associated deaths were reported in any EC. Next, we plotted an epidemiological tree of confirmed influenza cases using data from EC (B) and (C), from where most influenza cases were reported (Figure 2). The familial link accounted for 40% of influenza cases in EC (B) and 32% of cases in EC (C). All familial links were observed within the same room and space except one. There were 16 and 24 rooms and spaces where the evacuees were housed in EC (B) and (C) respectively. Of them, none of cases were detected in 5 (31.2%) and 14 (58.3%) rooms and spaces in these ECs. Six rooms and spaces reported two or more influenza cases that accounted for 83% of the total cases in EC (B), whereas there were 6 rooms and spaces with ≥2 cases that accounted for 87% of all cases in EC (C). Thus, residing in the same room was an apparent risk factor followed by the familial link. Influenza cases were simultaneously identified across rooms and spaces within 1 week after identification of the index cases. Vaccination histories prior to the influenza season was only available among 32.0% of evacuees in EC (B) and (C), and 48.7% were not vaccinated. Cohabitation of identified cases to designated rooms in all ECs was implemented to prevent further transmission. All confirmed influenza cases were administered antiviral drugs. The implementation of personal protective measures such as wearing masks and maintaining hand hygiene using alcohol-based hand sanitizers or running water, if available, were repeatedly encouraged, and the majority of evacuees abided by these precautions.

**Table 1:** Basic profiles of five evacuation centres and influenza cases, Yamamoto town

**Evacuation centres**	No. of evacuees*	**% of ≤ 15 years in evacuees**	**% of ≥ 65 years in evacuees**	**No. of influenza case**	**M/F ratio**	**Index case (age, sex)**	**Mean age of influenza cases**	AR** (%)
Evacuation centre A	130	NA	NA	10	4/6	60s, M	54.4	7.7
Evacuation centre B	702	12.1	36.3	60	27/33	48, M	51.0	8.6
Evacuation centre C	606	10.1	28.7	31	16/15	54, M	50.7	5.1
Evacuation centre D	121	NA	NA	2	0/2	46, F	31.5	1.7
Evacuation centre E	251	NA	NA	2	2/0	10s, M	21.5	0.8

Note: *The numbers of evacuees in Evacuation Centre A, D, and E were reported on March 25, 2011. The number of evacuees in Evacuation Centre B and C were reported on March 21, 2011. **AR, attack rate.

## Discussion

An outbreak of influenza A (H3N2) occurred in the ECs after the Great East Japan Earthquake of 2011. Natural disasters, including earthquakes, may lead to a surge of evacuees, population displacement, and a breakdown of the healthcare system in affected areas. Consequently, the risk of an infectious disease outbreak after a natural disaster is increased because of various factors such as poor hygiene and overcrowding of emergency shelters [20]. A variety of infectious diseases can potentially cause outbreaks following natural disasters [6]; however, the source of infection is more likely to be a microbial agent circulating in the community rather than one introduced from the outside [8]. The Great East Japan Earthquake occurred on 11 March 2011 when influenza was still circulating in Japan; therefore, the possibility of an influenza outbreak at that time was increased as reported in a previous study [15]. The influenza outbreak began more than 1 week after the earthquake and simultaneously spread to 5 ECs and accounted for 105 evacuees in Yamamoto. ECs where higher AR was recorded had the adult male case as an index case and 51.7% and 71.0% of cases were aged between 15 and 64 years in EC (B) and (C) respectively. Although an exact transmission route was not identified, our findings suggested that the influenza virus was introduced into the ECs through search and rescue activities, particularly in the early phase of this outbreak, in which a large number of working age adults were engaged together with others arriving from affected areas. Previous molecular study observed different clades of influenza A (H3N2) before and after the earthquake [14]. Similar to the cholera outbreak after the Haitian earthquake in 2010, molecular evidence indicated that *Vibrio cholerae* was introduced from outside of the affected area [21-23]. Taken together, these findings indicated a potential risk of the introduction of infectious diseases into affected areas by relief workers. Thus, it is important to ensure the health status and vaccination history among relief workers before they are permitted to enter affected areas.

The AR in children <15 years old was estimated to be less than the average AR in both EC (B) and (C), but this inding did not directly reflect a low proportion of children in the ECs because the proportions were compatible with those reported in the 2010 National Census. In fact, children are more frequently in contact with their parents and relatives than schoolmates or playmates as indicated by a study regarding social mixing during school holidays [24]. In our study, the majority of childhood cases had either a room link or a familial link [80% in EC (B) and 67% in EC (C)]. Other than these two ECs, there was one influenza case each in EC (A) and EC (E), in which the patient was <15 years. The latter case was the index case, but the route of infection remained unclear.

There were no deaths reported in this outbreak; however, the population of ≥65 years accounted for 21.9% cases. Taken together with the fact that influenza A (H3N2) cases are more severe, particularly in the elderly [25], this outbreak had the potential to cause significant morbidity and mortality, which motivated healthcare officials in Japan to prioritize measures to prevent the spread of influenza in ECs and to promote early treatment, particularly in the elderly. Finally, the mean AR was 5.3% (range, 0.8%–11.1%), which was relatively low, indicating successful interruption of influenza transmission by implementing several preventive measures such as active case identification, case cohorting in designated rooms, and wearing masks as previously described [15].

This study had some limitations. First, we could only obtain 27 POC residues from the 105 cases and 22 were positive for influenza A (H3N2). However, a surveillance study revealed a predominant circulation of influenza A(H3N2) in ECs in Miyagi Prefecture [11]. We believe that the observed outbreaks were caused by influenza A(H3N2). Second, we could only obtain background information of the evacuees in two ECs; therefore, we may have missed some characteristics of influenza transmission, particularly in the ECs with only two cases. Third, information regarding control measures such as wearing masks and vaccination history was insufficient; therefore, it was somewhat difficult to evaluate the effectiveness of control measures in the ECs. In spite of these limitations, a combination of preventive measures consisting of case cohorting, personal hygiene, wearing masks and early detection and treatment led to a low influenza AR.

## Conclusions

In conclusion, influenza can cause outbreaks in a disaster setting when the disaster occurs during an epidemic influenza season. The transmission route is more likely to be associated with same-room and familial links. The importance of monitoring influenza in ECs for implementing preventive control measures should be emphasized.

## Competing interest

The authors declare that they have no competing interests.

## Authors’ contributions

TK conceived of the study and participated in its design, carried out the statistical analysis and drafted the manuscript. JS participated in its design and collect the data. KT, NNS and KO collected the data and performed PCR tests. HO participated in its design and helped to draft the manuscript. All authors read and approved the final manuscript.

## Pre-publication history

The pre-publication history for this paper can be accessed here:

http://www.biomedcentral.com/1471-2458/14/34/prepub
